# Starting a New Aging Center in Uncertain Times: Recommendations and Lessons Learned

**DOI:** 10.1093/geroni/igaf122.610

**Published:** 2025-12-31

**Authors:** Pamela Saunders

**Affiliations:** Georgetown University, Washington, District of Columbia, United States

## Abstract

The national landscape poses significant challenges for the future of socio-biomedical research in the US. As a result, aging centers are evaluating their programs, services, structures, and sources of support. This paper discusses the launch of a new Center for Healthy Aging at Georgetown University Medical Center (GUMC), detailing its leadership, operations, and activities. GUMC includes schools of medicine, health, and nursing. While many faculty members conducted research on aging, there was no central unit to promote collaboration and networking across schools. To start, the Dean of Research formed a working group to strategize the future of aging research, resulting in a proposal for a new center. It was subsequently submitted to and approved by the Medical Center Committee on Research. The leadership structure includes a steering committee, a director, and an associate director. Members of the steering committee were invited to serve by the Dean of Research from the original working group and were primarily researchers. Later, additional faculty members were invited to join to develop educational resources and community partnerships. The Center’s operations involve monthly meetings to discuss activities and priorities. The Center hosts an annual Symposium featuring speakers from NIH, Georgetown, and local universities. Panel topics included social determinants of health, cognitive aging, and cancer in relation to aging. The Center’s future goals include conducting a SWOT analysis to establish priorities, submitting a conference grant to broaden national participation, and formalizing funding sources for programs and activities. Recommendations and lessons learned will be discussed.

